# Splicing of Friend Murine Leukemia Virus *env*-mRNA Enhances Its Ability to Form Polysomes

**DOI:** 10.3389/fmicb.2016.00160

**Published:** 2016-02-16

**Authors:** Akihito Machinaga, Syuhei Ishihara, Akiko Shirai, Sayaka Takase-Yoden

**Affiliations:** ^1^Department of Bioinformatics, Graduate School of Engineering, Soka UniversityTokyo, Japan; ^2^Department of Science and Engineering for Sustainable Innovation, Faculty of Science and Engineering, Soka UniversityTokyo, Japan

**Keywords:** murine leukemia virus, *env*-mRNA, splicing, polysome, Env protein

## Abstract

Friend murine leukemia virus (MLV) belongs to the gamma retroviruses of the Retroviridae family. The positive-sense RNA of its genome contains a 5′ long terminal repeat (LTR), 5′ leader sequence, *gag*, *pol*, *env*, and 3′ LTR. Transcription from proviral DNA begins from the R region of the 5′ LTR and ends at the polyadenylation signal located at the R region of the other end of the 3′ LTR. There is a 5′ splice site in the 5′ leader sequence and a 3′ splice site at the 3′ end of the *pol* region. Both full-length unspliced mRNAs and a singly spliced mRNA (*env*-mRNA) are produced in MLV-infected cells. The MLV Env protein plays important roles both in viral adsorption to host cells and in neuropathogenic disease in MLV-infected mice and rats. Understanding the regulatory mechanisms controlling Env expression is important for determining the functions of the Env protein. We have previously shown that splicing increases *env*-mRNA stability and translation efficiency. Generally, mRNA polysome formation correlates with translation efficiency. Therefore, here we investigated the effects of *env*-mRNA splicing on polysome formation to identify mechanisms for Env up-regulation due to splicing. We performed polysome profile analyses using Env-expression plasmids producing spliced or unspliced *env*-mRNA and showed that the former formed polysomes more efficiently than the latter. Thus, splicing of *env*-mRNA facilitated polysome formation, suggesting that this contributes to up-regulation of Env expression. We replaced the *env* region of the expression plasmids with a *luciferase* (*luc*) gene, and found that in this case both unspliced and spliced *luc*-mRNA formed polysomes to a similar extent. Thus, we conclude that whether mRNA polysome formation is affected by splicing depends on the structure of gene in question.

## Introduction

Friend murine leukemia virus (MLV) is a gamma retrovirus, a member of the Retroviridae family. It has a positive-sense RNA genome containing a 5′ LTR, 5′ leader sequence, *gag*, *pol*, *env*, and a 3′ LTR. Proteins responsible for the constitution of the inner structures of the virion, the matrix, capsid, and nucleocapsid proteins, are encoded by the *gag* region. The *pol* region encodes the enzymatic proteins, i.e., the reverse transcriptase, protease, integrase, and RNase H, and the *env* region encodes the Env protein protruding out from the viral particle surface. The Env protein is synthesized as a precursor protein (gpr85), and subsequently the gpr85 is cleaved into two subunits, the surface (SU) and transmembrane (TM) proteins. After entry of viral nucleocapsid into cells, double-stranded viral-DNA is produced from viral genome RNA by reverse transcriptase, which associates with viral nucleocapsids. This viral DNA is integrated into the genome of host cells, and at this stage is referred to as a provirus. Transcription from this proviral DNA begins from the R region of the 5′ LTR and ends at the polyadenylation signal located at the R region at the other end of the 3′ LTR. There is a 5′ splice site (5′ss) in the 5′ leader sequence and a 3′ splice site (3′ss) at the 3′ end of the *pol* region. Both full-length unspliced mRNAs and a singly spliced mRNA are produced in MLV-infected cells. Gag and Pol proteins are translated from unspliced mRNAs but the Env protein is translated from spliced mRNA (Coffin et al., 1997). The MLV Env protein plays important roles in viral adsorption to host cells. Infection by ecotropic MLV, including the Friend MLV, is mediated by the binding of the viral Env protein to the rodent ortholog of cationic amino acid transporter 1 (CAT-1), which serves as the specific cellular receptor (Albritton et al., 1989; Kim et al., 1991; Wang et al., 1991). The MLV Env protein also contributes markedly to viral neuropathogenicity. Several MLVs cause spongiform neurodegeneration when neonatal mice and/or rats are infected (Gardner, 1978; Wong et al., 1983; Kai and Furuta, 1984; Bilello et al., 1986; Wong, 1990; Masuda et al., 1992; Czub et al., 1995; Takase-Yoden and Watanabe, 1997; Tanaka et al., 1998). Some uninfected neurons may exhibit cytopathogenicity, indicating an indirect mechanism of MLV-induced neuropathogenicity. One common feature of neuropathogenic MLVs, including the Friend MLV A8 strain (the isolate used in the present study), is that the primary determinant of the induction of neurodegenerative disease resides in the *env* region (DesGroseillers et al., 1984; Yuen et al., 1986; Szurek et al., 1988; Paquette et al., 1989; Masuda et al., 1992, 1993; Takase-Yoden and Watanabe, 1997). However, the pathomechanism of spongiosis caused by MLV is still not understood, and it has not been determined which properties of the Env protein contribute to neurodegeneration. Recently, it has been proposed that endoplasmic reticulum (ER) stress and/or oxidative stress induced by accumulation of the precursor protein of Env (gpr85) after inefficient processing or unstable protein folding in glial cells could be mechanisms responsible for neurodegeneration (Dimcheff et al., 2003; Kim et al., 2004; Qiang et al., 2004). In Friend MLV A8 infection, however, such an accumulation of gpr85 in brains has not been observed, but we have previously shown that the amount of the neuropathogenic A8-MLV Env protein in brains, which is cleaved normally, does correlate with neuropathogenicity (Takase-Yoden and Watanabe, 2005; Takase-Yoden et al., 2006). Thus, definition of the regulatory mechanisms responsible for Env expression is important for understanding the functions of the Env protein. We previously found that splicing of *env*-mRNA increases the amount of Env protein translated from each molecule of the *env*-mRNA (Yamamoto and Takase-Yoden, 2009). However, mechanisms responsible for the post-transcriptional up-regulation of Env expression as a result of splicing are still not clear.

Polysome formation of mRNA is a factor in the regulation of protein expression or translation efficiency. Multiple ribosomes can bind to an mRNA to form a polysome structure, enabling many ribosomes to progress along an mRNA simultaneously to synthesize the same protein. Generally, in eukaryotic cells, mRNAs bound to a cluster of ribosomes (polysomes) circularize primarily by interactions between the poly(A) binding protein and the translation initiation factor to bind the mRNA 5′ end (Imataka et al., 1998; McCarthy, 1998; Wells et al., 1998; Kahvejian et al., 2005). Recently, it was reported that spliced mRNAs yield greater quantities of protein per mRNA molecule than do otherwise identical mRNAs not made by splicing (Wiegand et al., 2003; Nott et al., 2004; Diem et al., 2007; Lee et al., 2009). As described above, our previous study also showed that splicing of *env*-mRNA of MLV promoted translational efficiency (Yamamoto and Takase-Yoden, 2009). As possible mechanisms for the enhancement of translational efficiency by splicing, it was shown that splicing increases the translational yield from expression vectors carrying the T cell receptor (TCR)-β gene or the β-globin gene and that this correlates with enhanced cytoplasmic polysome association of the spliced mRNAs (Nott et al., 2004). In addition, it is suggested that exon junction complexes (EJCs), which are complexes of host factors that bind to spliced mRNAs, are involved in mRNA polysome association. On the other hand, it seems that mRNAs can also form polysome structures independently of splicing. It has been shown that unspliced retroviral *gag*-encoded mRNA can form polysome structures (Bartels and Luban, 2014). However, for the MLV *env*-mRNA, correlations between splicing and polysome formation have not been clarified.

In the present study, we determined the effects of *env*-mRNA splicing on polysome formation by using Env expression plasmids that produced either spliced or unspliced *env*-mRNA. In this way, we were able to study mechanisms involved in Env up-regulation due to splicing. We found that splicing increased the formation of *env*-mRNA polysome structures. Interestingly, this splicing-dependent phenomenon was not observed with expression plasmids in which the *env* region was replaced by the *luc* gene. These observations contribute to our understanding of post-transcriptional regulation of Env expression levels in MLV and may apply generally to gamma retroviruses.

## Materials and Methods

### Cell Culture and Transfection

NIH3T3 cells were grown in Dulbecco’s Modified Eagle’s Medium – low glucose (Sigma–Aldrich) supplemented with 10% fetal calf serum (MP Biomedicals), 50 units penicillin (Gibco)/ml and 50 μg streptomycin (Gibco)/ml, at 37°C in 7% CO_2_. Cell samples (1 × 10^6^ cells) were seeded in 6-cm dishes containing growth medium without penicillin and streptomycin. The cells were transfected the next day with 8 μg viral or luciferase expression vectors using Lipofectamine 2000 Reagent (Invitrogen) diluted with Opti MEM (Invitrogen) according to the manufacturer’s instructions.

Construction of the plasmids used in this study was described previously (Yamamoto and Takase-Yoden, 2009). However, vectors m1, d4, and m1gpL were previously designated proA8m1, pA8d4, and proA8gpL, respectively. The m1 plasmid carries the full-length A8-MLV provirus genome (accession No. D88386). G to T (nt 2608), G to T (nt 2614), and G to T (nt 2629) mutations were introduced into the *pol* region in m1 and m1gpL. These mutations generate stop codons to inhibit Pol protein production and to suppress the production of infectious progeny virus, respectively. m1 and m1gpL also had the point mutations A to T (nt 2126 in the *gag* region) and T to A (nt 2777 in the *pol* region), which had occurred spontaneously during construction of the plasmids. They resulted in the amino acid changes Gln to Leu and Ile to Lys, respectively.

### Fractionation of Cell Lysates by Sucrose Density Gradient Centrifugation

Polysome fractions were obtained by fractionation of cell extracts by sucrose density gradient centrifugation as described previously (Esposito et al., 2010). Briefly, 48 h post-transfection, or after persistent viral infection, NIH3T3 cells (6 × 10^6^) were incubated in medium containing 100 μg cycloheximide/ml for 15 min. It was previously shown that 48 h post-transfection, *env*-mRNA and Env protein were sufficiently expressed for analysis (Yamamoto and Takase-Yoden, 2009). The cells were then lysed in 1 ml hypotonic lysis buffer [1.5 mM KCl, 2.5 mM MgCl_2_, 5 mM Tris-HCl (pH 7.4), 1% Triton X-100, 1% sodium deoxycholate, 100 μg cycloheximide/ml, 1 mM dithiothreitol, 100 units RNase inhibitor (TaKaRa)/ml]. After 10 min on ice, lysates were centrifuged at 10,000 × *g* for 10 min and the resulting cytosol-containing supernatant was removed and layered onto a 10–50% sucrose density gradient in buffer [80 mM NaCl, 5 mM MgCl_2_, 20 mM Tris-HCl (pH 7.4), 1 mM dithiothreitol]. After ultracentrifugation using an SW 41 rotor in an Optima XL-90 ultracentrifuge (Beckman) at 30,000 rpm for 3 h at 4°C, 16 fractions were obtained. The RNA in each fraction was extracted using TRIzol LS Reagent (Invitrogen) and measured by absorbance at 260 nm using Gene Quant pro (Amersham Biosciences).

### Detection of Ribosomal RNA

Extracted RNA from each fraction was denatured in sample buffer [50% (v/v) formamide, 1x MOPS (20 mM MOPS, pH 7.0, 8.3 mM NaAc, 1 mM EDTA), 15% (v/v) formaldehyde, 3.35% (v/v) ethidium bromide] and analyzed by electrophoresis on a 1% denaturing agarose gel. The 28S and 18S rRNAs were detected as ethidium bromide-stained bands on the gels.

### Quantification of *env*-, *luc*-, and *gapdh*-mRNA in Each Fraction by Real-Time RT-PCR

Extracted RNA from each fraction was treated with RNase-free DNase (QIAGEN). Equal volume samples of each fraction were used as templates for reverse transcription using an oligo(dT) primer (Invitrogen). Two microgram RNA was contained in its volume of the fraction that had the largest absorbance peak at 260 nm. A portion of the resulting cDNA was amplified by real-time PCR using a 7500 Real-Time PCR System (Applied Biosystems). The primers and probes used to quantitate *env*- and *luc*-mRNA were forward s1-primer, 5′-GAGACCCTTGCCCAGGGA-3′; reverse s2-primer, 5′-TGCCGCCAACGGTCTCC-3′; and TaqMan ss-probe, 5′-CACCACCGGGAGCTCATTTACAGGCAC-3′. These primers were designed to amplify the exon–exon junction region of *env*- and *luc*-mRNA. The primers and probe used to quantify i-*env*-mRNA, which was produced by the d4 vectors, were forward i1-primer, 5′-GGCGGACCCGTGGTAGA-3′; reverse i2-primer, 5′-GATTTCATACTCCCAGGGTTGC-3′; and TaqMan i-probe, 5′-CTGACGAGTTCGGGATACCCGGC-3′. These primers were designed to amplify the AatII–NdeI junction region of i-*env*-mRNA. To quantify *gapdh* mRNA, TaqMan Rodent GAPDH Control Reagents containing primer sets and probe (Applied Biosystems) were used. Standard curves used to calculate the amount of mRNA were produced using serial dilutions of expression plasmids and the gapdh T-easy vector containing a fragment of the rodent *gapdh* gene. Negative control samples without the cDNA synthesis step did not show specific amplification. Statistical comparison was done using the *t*-test.

## Results

### Formation of Polysome Structures of Spliced *env*-mRNA

**FIGURE 1:**
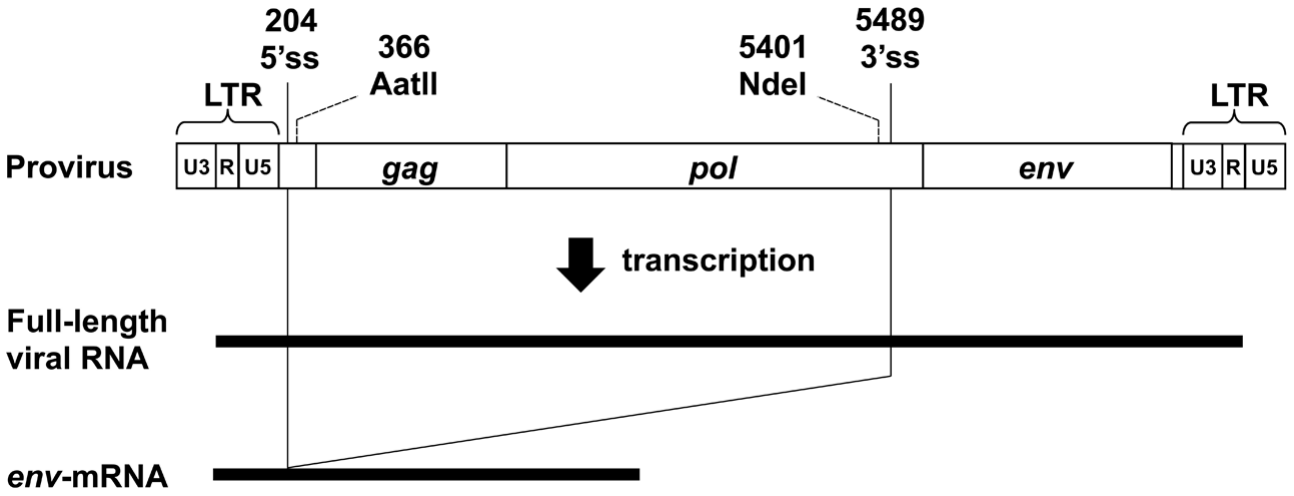
**Structure of the MLV provirus genome and its transcripts.** 5′ss, 5′ splice site; 3′ss, 3′ splice site. The numbering of nucleotides is based on the transcript. The *Aat*II recognition site and *Nde*I recognition site were used for construction of the d4 plasmid (see **Figure 3**).

**FIGURE 2:**
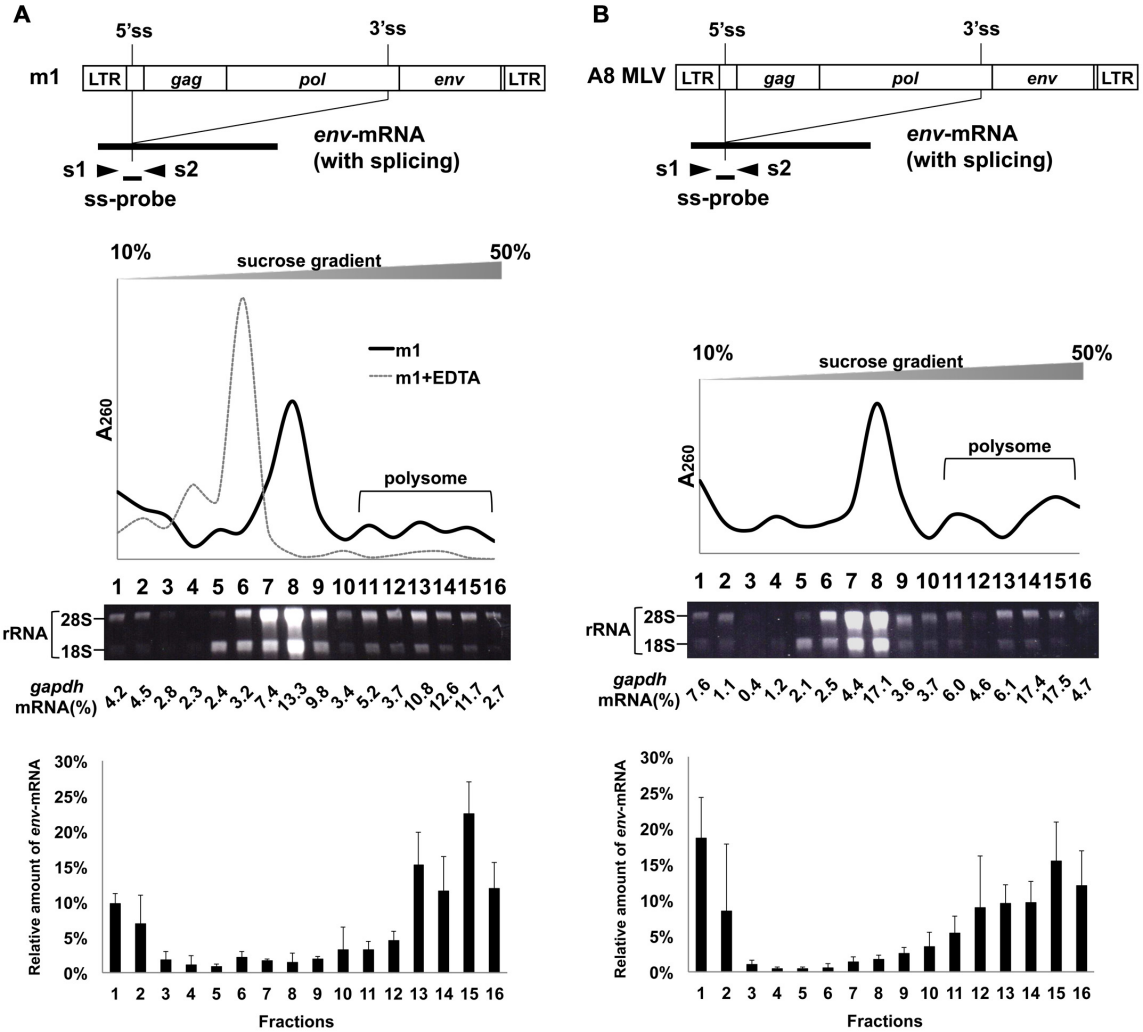
**Polysome profiles of cells transfected with m1 and infected with MLV.** Fractionation of ribosomes from cells **(A)** transfected with m1 and **(B)** infected with MLV A8 strain. G to T (nt 2608), G to T (nt 2614), and G to T (nt 2629) mutations were introduced into the *pol* region in m1. These mutations create stop codons to inhibit Pol protein production and to suppress the production of infectious progeny virus, respectively. m1 also had point mutations A to T (nt 2126 in the *gag* region) and T to A (nt 2777 in the *pol* region). These mutations had occurred spontaneously during construction of the plasmids and caused amino acid changes Gln to Leu and Ile to Lys, respectively. Cell lysates were centrifuged through a 10–50% sucrose density gradient and fractionated, and RNA was extracted from each fraction. Ribosomal RNA was analyzed by electrophoresis on a 1% denaturing agarose gel. To quantify *gapdh* mRNA, TaqMan Rodents GAPDH Control Reagents containing primer sets and probe were used. The results of the distribution of total RNA, ribosomal RNA, and *gapdh*-mRNA are representative of three or four experiments, with similar results. For the cell lysate after treatment with 25 mM EDTA, the results of the distribution of total RNA are presented. The amount of *env*-mRNA was measured by real-time RT-PCR using s1 and s2 primers and ss-probe, and the amount in each fraction relative to the total amount of *env*-mRNA in all fractions was calculated. The bottom graphs show the *env*-mRNA distribution from 3 or 4 independent experiments, with the mean ± standard error of each fraction.

The MLV genome has a 5′ss in the 5′ leader sequence and a 3′ss at the 3′ end of the *pol* region. Both full-length unspliced mRNA and singly-spliced *env*-mRNA are produced from the provirus (**Figure 1**). We have previously shown that splicing of *env*-mRNA enhances translation efficiency of the Env protein (Yamamoto and Takase-Yoden, 2009). Generally, polysome formation of mRNA is correlated with mRNA translation efficiency. To investigate whether spliced *env*-mRNA forms polysome structures, we used the m1 plasmid which carries the full-length A8-MLV provirus genome and generates spliced *env*-mRNA (**Figure 2A**). NIH3T3 cells were transfected with this plasmid, and cell lysate was separated by centrifugation on linear 10–50% sucrose density gradients, as described in the Materials and Methods. After extraction of RNA from each fraction, the distribution of total RNA and ribosomal RNA (rRNA) was analyzed by measurement of absorbance at 260 nm and agarose gel electrophoresis, respectively (**Figure 2A**). This approach yields mRNA in polysome structures in the higher density fractions, while mRNA not in polysome structures is in the lower density fractions, as reported previously (Otulakowski et al., 2004; Nashchekin et al., 2006; Akimitsu et al., 2007; del Prete et al., 2007; Holetz et al., 2007; Ryu et al., 2008). Here we found that there was a major peak of absorbance at 260 nm in fractions 6–9 of lysates of m1-transfected cells (**Figure 2A**). Agarose gel electrophoresis showed that these fractions contained most of the 28S and 18S rRNAs in the lysate. There were small peaks at 260 nm in the higher density fractions 11–16, which also contained 28S and 18S rRNA, consistent with these fractions containing polysomes as well. The distribution of *gapdh*-mRNA was established by real-time RT-PCR as a control, yielding two peaks, one in lower density fraction 8 and the other in higher density fractions 13-15 (**Figure 2A**). These results are in agreement with a previous report (Akimitsu et al., 2007). In addition, to confirm that this observed distribution pattern of RNA is attributable to ribosome association, we tested the effects of EDTA treatment, as described previously (Henshaw, 1968; del Prete et al., 2007). Throughout the polysome profiling procedure, magnesium was added to the lysis buffer and the gradient buffer to stabilize 80S ribosomes. Sequestering magnesium ions with EDTA added to the regents causes separation of the ribosome into 40S and 60S subunits. Thus, if absorbance peaks recorded at 260 nm are indeed polysomes, EDTA treatment will collapse the profile such that a single maximum will be observed near the top of gradient that corresponds to free mRNA and ribosomal units (Faye et al., 2014). After treatment of cell lysate with 25 mM EDTA, the distribution pattern shifted toward the lighter sucrose fractions as shown in **Figure 2A**, indicating that the mRNA had dissociated from polysomes. The distribution of *env*-mRNA was examined by real-time RT-PCR using s1 and s2 primers and the ss-probe which recognizes the splice junction region of *env*-mRNA. As shown at the bottom of **Figure 2A**, most *env*-mRNA was found in fractions 13–16, i.e., the polysome fractions. From quantitative analysis of these real-time PCR results, we conclude that 69% of the *env*-mRNA in m1-transfected cells was in polysomes in fractions 11–16 (**Table 1**). A similar experimental analysis of lysates of NIH3T3 cells infected with A8-MLV (**Figure 2B**) showed that 61% of *env*-mRNA was in polysome fractions 11–16 in these cells, not significantly different from the 69% in m1-transfected cells (**Table 1**). These results document that spliced *env*-mRNA forms polysome structures.

**Table 1:** Relative amount of *env*-mRNA and *luc*-mRNA in polysome fractions.

Virus or plasmid	Structure of mRNA	mRNA spliced	Relative amount of mRNA in polysomes (%)^a^
m1	*env*-mRNA	yes	69 ± 6.1^b^
MLV A8	*env*-mRNA	yes	61 ± 7.8
splA8	*env*-mRNA	no	24 ± 4.0^c^
d4	*env*-mRNA	yes	69 ± 2.1
	i-*env*-mRNA	no	30 ± 5.0^d^
m1gpL	*luc*-mRNA	yes	68 ± 1.1
splA8L	*luc*-mRNA	no	60 ± 2.6^e^

^a^*The mean values from 3 to 4 independent experiments with standard errors are shown. Statistical comparisons were performed using the t-test.*^b^*No significant difference compared to A8 virus.*^c^*Significant difference (p < 0.01) compared to m1.*^d^*Significant difference (p < 0.01) compared to d4 *env*-mRNA.*^e^*No significant difference compared to m1gpL*.

### Effects of Splicing on Polysome Formation of *env*-mRNA

**FIGURE 3:**
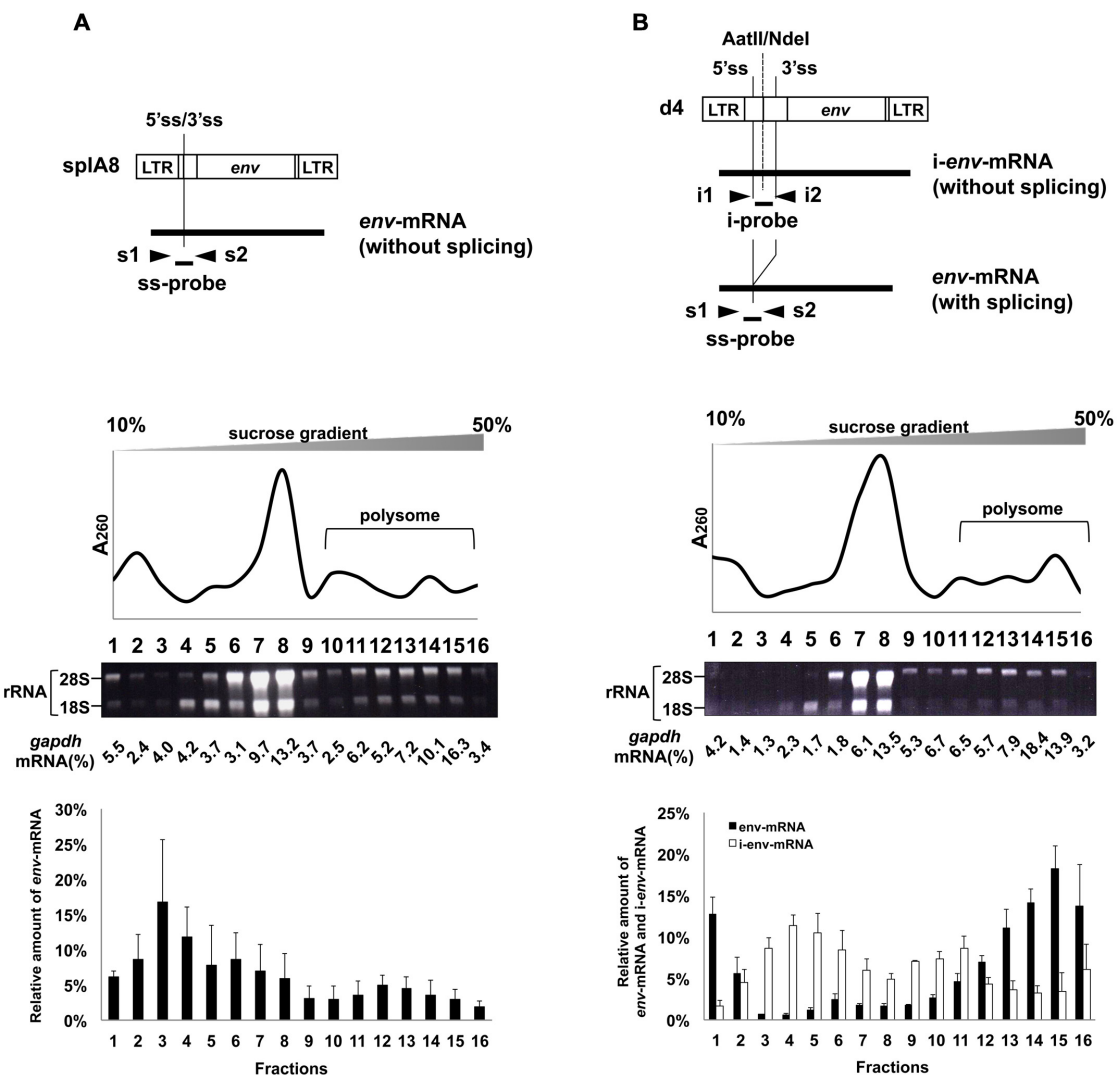
**Polysome profiles of cells transfected with splA8 and d4.** Fractionation of ribosomes from cells transfected with **(A)** splA8 and **(B)** d4. The splA8 plasmid generated unspliced *env*-mRNA. The d4 plasmid normally produced both spliced transcripts (*env*-mRNA) and unspliced transcripts (i-*env*-mRNA). The amount of i-*env*-mRNA was measured by real-time RT-PCR using i1 and i2 primers and an i-probe. Polysome profile analyses were performed using splA8- and d4-transfected cells. The bottom graphs show the *env*-mRNA and i-*env*-mRNA distribution from 3 or 4 independent experiments, with the mean ± standard error of each fraction.

To investigate whether splicing of *env*-mRNA affected its ability to form polysomes, we used the splA8 plasmid (Yamamoto and Takase-Yoden, 2009), which was designed to generate unspliced *env*-mRNA by deletion of the intron region in m1 (**Figure 3A**). NIH3T3 cells were transfected with splA8, and the cell lysates separated by centrifugation on linear 10–50% sucrose density gradients as before. A large amount of *env*-mRNA was found in fractions 1–8 in lysates of these cells (bottom graph of **Figure 3A**). Although 24% of *env*-mRNA in splA8-transfected cells was in polysomes (fractions 10-16), this was significantly less (*p* < 0.01) than the 69% in m1-transfected cells (**Table 1**). Very similar results were obtained with a different cell type, RS-A. These are spontaneously transformed cells derived from rat spleen (Tokiwa, 1972). Here, the amount of *env*-mRNA-associated polysome fractions in splA8-transfected cells was also significantly lower than in m1-transfected cells (data not shown).

We also analyzed the distribution of *env*-mRNA in d4-transfected cells to compare the amount of polysomes associated with spliced or unspliced mRNA. The d4 plasmid was constructed by deleting the AatII-NdeI fragment in m1 to generate a plasmid that produces both spliced and unspliced *env*-mRNA (i-*env*-mRNA) in transfected cells (**Figures 1** and **3B**; Yamamoto and Takase-Yoden, 2009). Because spliced *env*-mRNA and unspliced i-*env*-mRNA can be distinguished in d4-transfected cell lysates by real time RT-PCR, we were able to compare the distribution of these two RNAs in the same cells. In d4-transfected cells, we found that 69% of *env*-mRNA was in the polysome fractions 11–16 (**Figure 3B**; **Table 1**), whereas only 30% of i-*env*-mRNA was in those fractions, but a larger proportion was present in fractions 2–7. Therefore, we conclude that in d4-transfected cells, the fraction of spliced *env*-mRNA found in polysomes was significantly greater than the fraction of unspliced i-*env*-mRNA in polysomes (*p* < 0.01). These results confirmed that splicing of *env*-mRNA facilitated polysome formation.

### Effects of Splicing on Polysome Formation of *luc*-mRNA

**FIGURE 4:**
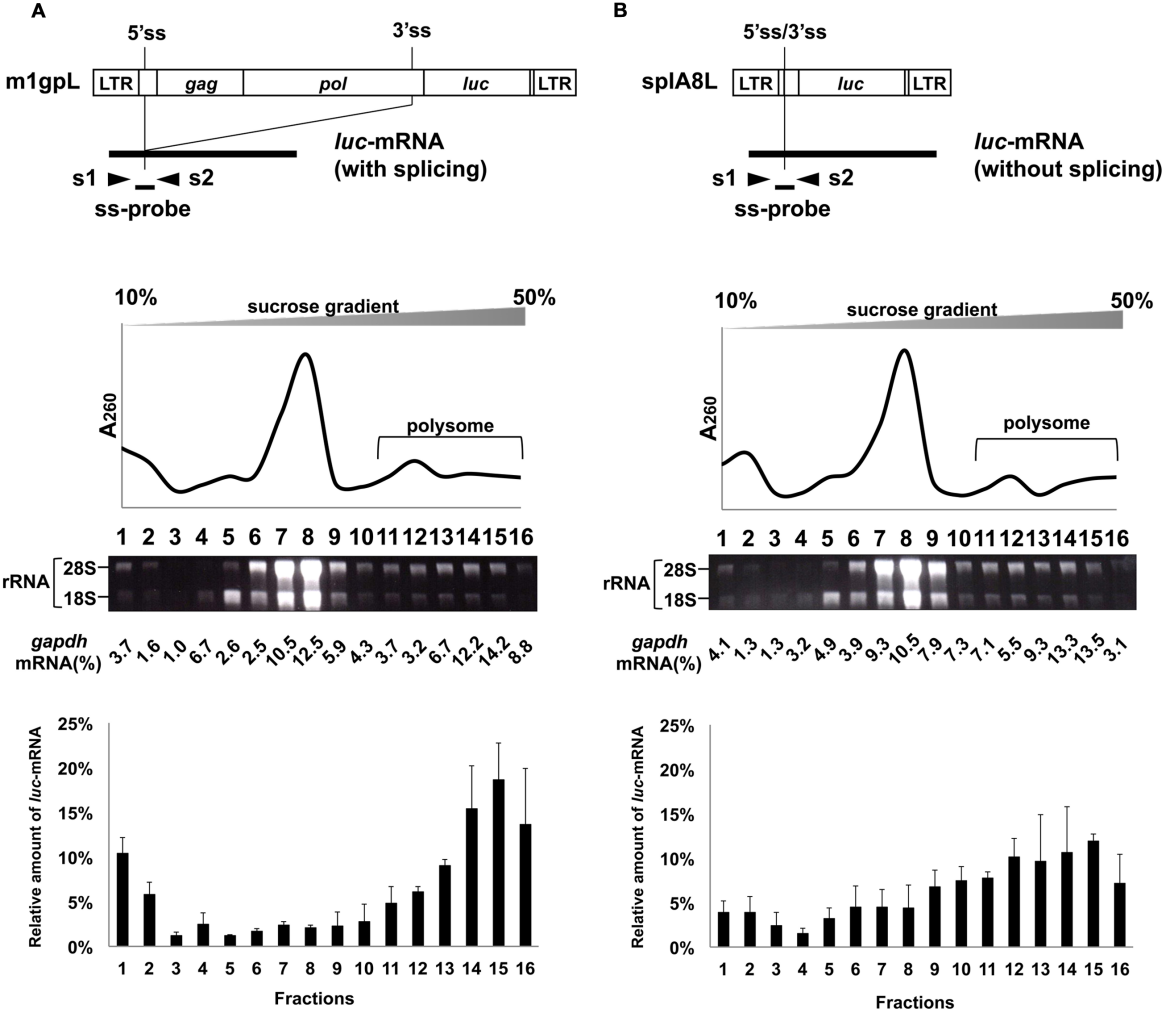
**Polysome profiles of cells transfected with m1gpL and splA8L.** Fractionation of ribosomes from cells transfected with **(A)** m1gpL and **(B)** sp1A8L. G to T (nt 2608), G to T (nt 2614), and G to T (nt 2629) mutations were introduced into the *pol* region in m1gpL. These mutations create stop codons to inhibit Pol protein production, respectively. m1gpL also had point mutations A to T (nt 2126 in the *gag* region) and T to A (nt 2777 in the *pol* region). These mutations had occurred spontaneously during construction of the plasmids and caused amino acid changes Gln to Leu and Ile to Lys, respectively. The amount of cDNA from *luc*-mRNA was measured by real-time RT-PCR using s1 and s2 primers and ss-probe. Polysome profile analyses were performed using m1gpL- and sp1A8L-transfected cells to express *luc*-mRNA. The bottom graphs show the *luc*-mRNA distribution from 3 independent experiments, with the mean ± standard error of each fraction.

Using m1gpL and splA8L plasmids in which the *env* gene in m1 and splA8 was replaced by the *luc* gene (**Figures 4A,B**), we previously showed that splicing of *luc*-mRNA did not influence its translation efficiency (Yamamoto and Takase-Yoden, 2009). Here, we analyzed the effect of splicing of *luc*-mRNA on polysome formation in cells transfected with m1gpL or splA8L. In the former, 68% of *luc*-mRNA was found in polysome fractions 11–16 and in the latter this was 60% (**Figure 4**; **Table 1**). Thus, there was no significant difference in the amounts of *luc*-mRNA in polysomes, showing that for this gene product, splicing did not affect polysome formation.

## Discussion

Our previous study showed that splicing of MLV *env*-mRNA increased its translation efficiency (Yamamoto and Takase-Yoden, 2009). In the present study, to investigate the mechanisms responsible for this up-regulation of Env expression, we investigated correlations between splicing of *env*-mRNA and its ability to form polysomes. We found that the fraction of spliced *env*-mRNA in polysomes was significantly greater than for unspliced *env*-mRNA (**Figures 2** and **3**). Thus, splicing promoted polysome formation of *env*-mRNA, thereby contributing to up-regulation of Env protein expression. To the best of our knowledge, this is the first report showing that splicing of a viral mRNA increased its ability to form polysomes. Nott et al. have reported that mRNAs of the TCR-β gene and the β-globin gene formed more polysome structures when expressed from vectors containing their introns than when expressed from vectors with truncated introns (Nott et al., 2004). It has also been suggested that EJCs promote mRNA polysome association. It is known that during pre-mRNA splicing in the nucleus, several proteins bind to a region 20-24 nucleotides upstream of mRNA exon–exon junctions to form EJCs (Le Hir et al., 2000; Reichert et al., 2002; Tange et al., 2004; Le Hir and Andersen, 2008; Schmidt et al., 2009), which are then transported with the mature mRNA to the cytoplasm and remain associated with the mRNA-binding proteins until the mRNA is translated. They are involved in different cellular processes, including nucleocytoplasmic mRNA export, subcellular localization, quality control, and translation (Kim et al., 2001; Le Hir et al., 2001; Dostie and Dreyfuss, 2002; Lejeune et al., 2002; Tange et al., 2004; Kashima et al., 2006; Giorgi and Moore, 2007; Le Hir and Seraphin, 2008). It has been proposed that EJCs may promote formation of polysome structures, thereby enhancing translation (Wiegand et al., 2003; Nott et al., 2004; Diem et al., 2007; Lee et al., 2009). Diem et al. reported that a 29 kDa protein, PYM, that binds EJC proteins in the cytoplasm also binds, via a separate domain, to the 40S ribosomal subunit and the 48S preinitiation complex. These investigators suggested that PYM functions as a bridge between EJC-associated spliced mRNAs and the translation machinery to enhance mRNA translation (Diem et al., 2007). Therefore, for MLV mRNA, it is possible that only spliced *env*-mRNA can associate with EJCs and form polysome structures, whereas unspliced *env*-mRNA cannot. Further experiments are needed to reveal the contribution of host factors that promote *env*-mRNA polysome formation due to splicing. On the other hand, the relative amounts of spliced *env*-mRNA and *luc*-mRNA in the lower density fractions 1 and/or 2 were also slightly higher compared to unspliced *env*-mRNA or i-*env*-mRNA (**Figures 2** and **3**) and unspliced *luc*-mRNA (**Figure 4**). It is likely that these fractions contained mRNAs that were free from ribosomes. It is possible that in the process of splicing, insufficiently processed mRNAs, which cannot associate with ribosomes, might be produced to a minor extent.

It was reported that the nuclear export receptor NXF1 (nuclear RNA export factor 1) is involved in nuclear export of RNA transcripts, especially unspliced mRNA, of gamma retroviruses including the xenotropic MLV-related virus (XMRV) and MLV (Sakuma et al., 2014). A conserved *cis*-acting element was identified at the 3′ end of the *pol* region of gamma retroviruses, designated the CAE (cytoplasmic accumulation element). Another NXF1-responsive element was identified within the 5′ half of the *pol* region, designated the gamma-CTE (*cis*-acting constitutive transport element; Bartels and Luban, 2014). Interestingly, it has been shown that recruitment of NXF1 into the gamma-CTE promotes polysome formation of unspliced *gag*-encoded mRNA (Bartels and Luban, 2014). However, it is unlikely that the gamma-CTE is involved in polysome formation of *env*-mRNA, because it is located at the intron which *env*-mRNA does not have. On the other hand, in MLV A8, *env*-mRNA does have a complete CAE region (Machinaga and Takase-Yoden, 2014). Although unspliced *env*-mRNA derived from the splA8 plasmid also has a complete CAE region, the level of polysome formation by its unspliced *env*-mRNA was low (**Figure 3**). Therefore, we suggest that the CAE region, which is an NXF1-responsive element, is not involved in polysome formation of *env*-mRNA.

Interestingly, when the *env* gene in m1 and splA8 was replaced by the *luc* gene, unspliced *luc*-mRNA formed polysome structures to a similar extent as the spliced variety (**Figure 4**). We have previously shown that there is no significant difference in the translation efficiency of spliced and unspliced *luc*-mRNA (Yamamoto and Takase-Yoden, 2009). Therefore, for *luc*-mRNA, the level of polysome formation and translation efficiency does not depend on splicing. The results also suggest that whether mRNA polysome formation is affected by splicing depends on the structure of the genes examined. The reason why unspliced *luc*-mRNA is able to form polysome structures is not understood. In the case of unspliced *gag*-encoded mRNA, it has been suggested that a shuttling protein, SRp20, which usually binds to NXF1 and is involved in mRNA export, also directly binds to this mRNA and promotes polysome formation through NXF1 (Bartels and Luban, 2014). It is possible that these host factors may contribute to polysome formation of unspliced *luc*-mRNA through the luciferase coding region. Further analyses are needed to determine the contribution of host factors promoting polysome formation of unspliced *luc*-mRNA.

In our previous study, we showed that a high level of expression of A8-Env protein in brains contributed to neuropathogenicity (Takase-Yoden and Watanabe, 2005; Takase-Yoden et al., 2006). However, a correlation between polysome formation of *env*-mRNA and neuropathogenicity of MLV is not clear in the present study. As discussed above, it is probable that the cellular factors involved in splicing are important for *env*-mRNA polysome formation. Therefore, it seems to be important for neuropathogenicity of MLV that these cellular factors are abundant in viral infected cells that commit to induction of neurodegeneration. In addition, because neuropathogenicity of MLV also depends on the sequence of the *env* gene, it is possible that unknown *cis*-elements that interact with these cellular factors might exist in the *env* region of neuropathogenic MLV but not in the *env* region of non-neuropathogenic MLV. To determine whether polysome formation of *env*-mRNA differs between neuropathogenic and non-neuropathogenic MLVs actually, further experiments are needed.

In summary, this study showed that splicing of *env*-mRNA facilitated polysome formation and thus was likely to contribute to the up-regulation of Env protein expression. However, when the *env* gene of the expression plasmids was replaced by the *luc* gene, unspliced *luc*-mRNA formed polysome structures to a similar extent as spliced *luc*-mRNA. These results indicate that whether mRNA polysome formation is affected by splicing depends on the structure of the genes studied.

## Author Contributions

AM performed experiments, analyzed the data, and wrote the manuscript. SI and AS performed experiments and analyzed the data. ST-Y conceived and designed the work, analyzed the data, and wrote the manuscript.

## Conflict of Interest Statement

The authors declare that the research was conducted in the absence of any commercial or financial relationships that could be construed as a potential conflict of interest.
